# Influence of IOL Weight on Long-Term IOL Stability in Highly Myopic Eyes

**DOI:** 10.3389/fmed.2022.835475

**Published:** 2022-04-11

**Authors:** Yuxi Chen, Jiaqi Meng, Kaiwen Cheng, Qiang Lu, Ling Wei, Yi Lu, Xiangjia Zhu

**Affiliations:** ^1^Department of Ophthalmology and Eye Institute, Eye and Ear, Nose, and Throat Hospital of Fudan University, Shanghai, China; ^2^Key Laboratory of Myopia (Fudan University), National Health Commission, Shanghai, China; ^3^Key Laboratory of Myopia, Chinese Academy of Medical Science, Shanghai, China; ^4^Shanghai Key Laboratory of Visual Impairment and Restoration, Shanghai, China; ^5^State Key Laboratory of Medical Neurobiology, Fudan University, Shanghai, China

**Keywords:** cataract surgery, high myopia, long-term, decentration, IOL weight

## Abstract

**Purpose:**

This study aimed to investigate the influence of intraocular lens (IOL) weight on long-term IOL stability in highly myopic eyes.

**Materials and Methods:**

A total of 205 highly myopic cataract eyes of 205 patients implanted with the MC X11 ASP (Group A, 86 eyes) or 920H IOL (Group B, 119 eyes) were included in this retrospective study. Eyes were divided into 3 subgroups according to the IOL power: low (≥-5 to <5 D), medium (≥5 to <14 D), and high (≥14 D) IOL power. At 3 years after surgery, IOL decentration and tilt, high-order aberrations, and anterior capsular opening (ACO) area were measured. The influence of IOL weight on long-term IOL stability was evaluated.

**Results:**

Group B had a significantly greater IOL weight than Group A (Group B vs. Group A: 28.31 ± 2.01 mg vs. 25.71 ± 4.62 mg, *P* < 0.001). Correspondingly, Group B presented significantly greater overall and inferior decentration than Group A, especially for low and medium IOL power (all *P* < 0.05). In both groups, overall and vertical decentration was significantly correlated with IOL weight (all *P* < 0.05). Group B showed a significantly greater ACO area than Group A (*P* < 0.05). Multivariate analysis showed that decentration in Group A was affected by IOL weight, while decentration in Group B was affected by IOL weight and AL.

**Conclusions:**

Higher IOL weight may lead to greater long-term IOL decentration in highly myopic eyes, while the haptic design may play a role in anterior capsular contraction.

## Introduction

The world is going myopic (1), especially during the COVID-19 pandemic lockdown, wherein the proportions of myopia and high myopia in children and teenagers increased according to the study of Qu et al. (2). In recent years, cataract surgery has become more of refractive surgery, geared more toward pursuing ideal visual quality instead of visual acuity restorations (3, 4). With the advancement of surgical techniques and materials, expectations for surgical outcomes are constantly increasing. Nonetheless, the number of highly myopic cataract patients also increased rapidly with the high prevalence of high myopia (1, 2). However, due to their unique anatomical structure of eyes (5), highly myopic patients are often confronted with more complications after cataract surgery compared to emmetropic patients (6).

The intraocular lens (IOL) malposition is a common post-operative complication of highly myopic cataract eyes (7, 8). As the lens capsular bag becomes larger with the elongation of axial length (AL), post-operative IOL decentration and tilt are found to be much more severe in highly myopic eyes, leading to worse visual quality (9–12). The MC X11 ASP (HumanOptics AG, Erlangen, Germany) and 920H (Rayner Intraocular Lenses Ltd., West Sussex, England) IOLs are two commonly used IOLs in highly myopic eyes. According to our clinical experience, the MC X11 ASP IOL tends to show better long-term stability compared to 920H in highly myopic eyes. A distinguished difference between the two IOLs is that the IOL weight varies widely within the same range of the IOL power. Considering that highly myopic eyes often show weaker zonular strength (9), we speculated that the IOL weight may affect the long-term IOL stability in highly myopic eyes after surgery. However, the relevant studies were rare.

The purpose of this study was to investigate the influence of IOL weight on long-term IOL stability by comparing the 3-year post-operative IOL stability between two monofocal IOLs (MC X11 ASP and 920H) in highly myopic eyes.

## Materials and Methods

This retrospective study was conducted at the Eye and Ear, Nose, and Throat Hospital, Fudan University, Shanghai, in accordance with the tenets of the Declaration of Helsinki. All procedures were approved by the institutional review board of the Eye and Ear, Nose, and Throat Hospital. Signed informed consents were obtained from all participants before cataract surgery for the use of their clinical data. The study was affiliated with the Shanghai High Myopia Study (registered at www.clinicaltrials.gov, accession number: NCT03062085).

### Patients

This study was done on humans. We reviewed the medical records of highly myopic patients (AL ≥ 26 mm) who underwent uneventful cataract surgery and implantation of MC X11 ASP or 920H IOLs (between January 2017 and April 2018) and completed 3-year post-operative follow-up. The exclusion criteria were the following: (1) presence of any other oculopathy, such as intraoperative floppy iris syndrome, pseudoexfoliation, corneal diseases, strabismus, uveitis, or glaucoma; (2) prior intraocular procedures or trauma; (3) severe intraoperative or post-operative complications, such as posterior capsule rupture or failure of continuous circular capsulorhexis; (4) pupil diameter of <6 mm after sufficient dilation. A total of 205 highly myopic eyes of 205 patients were included in the study, which were divided into the following groups: 86 eyes in Group A (MC X11 ASP) and 119 eyes in Group B (920H). For both groups, eyes were further classified into 3 subgroups according to the implanted IOL power: ≥-5 to <5 D (low IOL power), ≥5 to <14 D (medium IOL power), and 14 D or greater (high IOL power).

### Pre-Operative Examinations

Prior to surgery, all patients underwent complete ophthalmic examinations including assessment of uncorrected visual acuity [UCVA; logarithms of the minimal angle of resolution (logMAR)] and best-corrected visual acuity (BCVA; logMAR), slit-lamp examination, corneal topography (Pentacam HR, Oculus Optikgeraete GmbH, Wetzlar, Germany), AL measurements (IOLMaster-500, Carl Zeiss AG, Oberkochen, Germany), fundoscopy, B-scan ultrasonography, and optical coherence tomography (OCT, Zeiss Cirrus HD-OCT 5000; Carl Zeiss AG, Oberkochen, Germany). IOL power was calculated using the Haigis formula. The weights of the two IOLs were obtained from the corresponding manufacturers.

### Surgical Procedures

All surgeries were performed by a single, experienced surgeon (Prof. YL) following a standard procedure. A 2.6 mm temporal clear corneal incision was made, which was followed by a 5.5 mm continuous curvilinear capsulorhexis, hydrodissection, and phacoemulsification. The IOL was inserted into the capsular bag and aligned with the center. After thorough removal of the viscoelastic from above and below the IOL, the IOL position was reconfirmed, and then the incision was hydrated. As post-operative treatments for all patients, topical prednisolone acetate (Allergan Pharmaceutical Ireland, Westport, Ireland), and levofloxacin (Cravit, Santen Pharmaceutical, Shiga, Japan) were prescribed 4 times daily for 2 weeks and pranoprofen eyedrops (Pranopu- lin, Senju Pharmaceutical, Osaka, Japan) 4 times daily for 4 weeks.

### 3-Year Post-Operative Follow-Up

Three years after surgery, all patients underwent ophthalmic examinations, including assessment of UCVA and BCVA, OPD-scan examination, and slit-lamp anterior segment photography.

**Figure 1:**
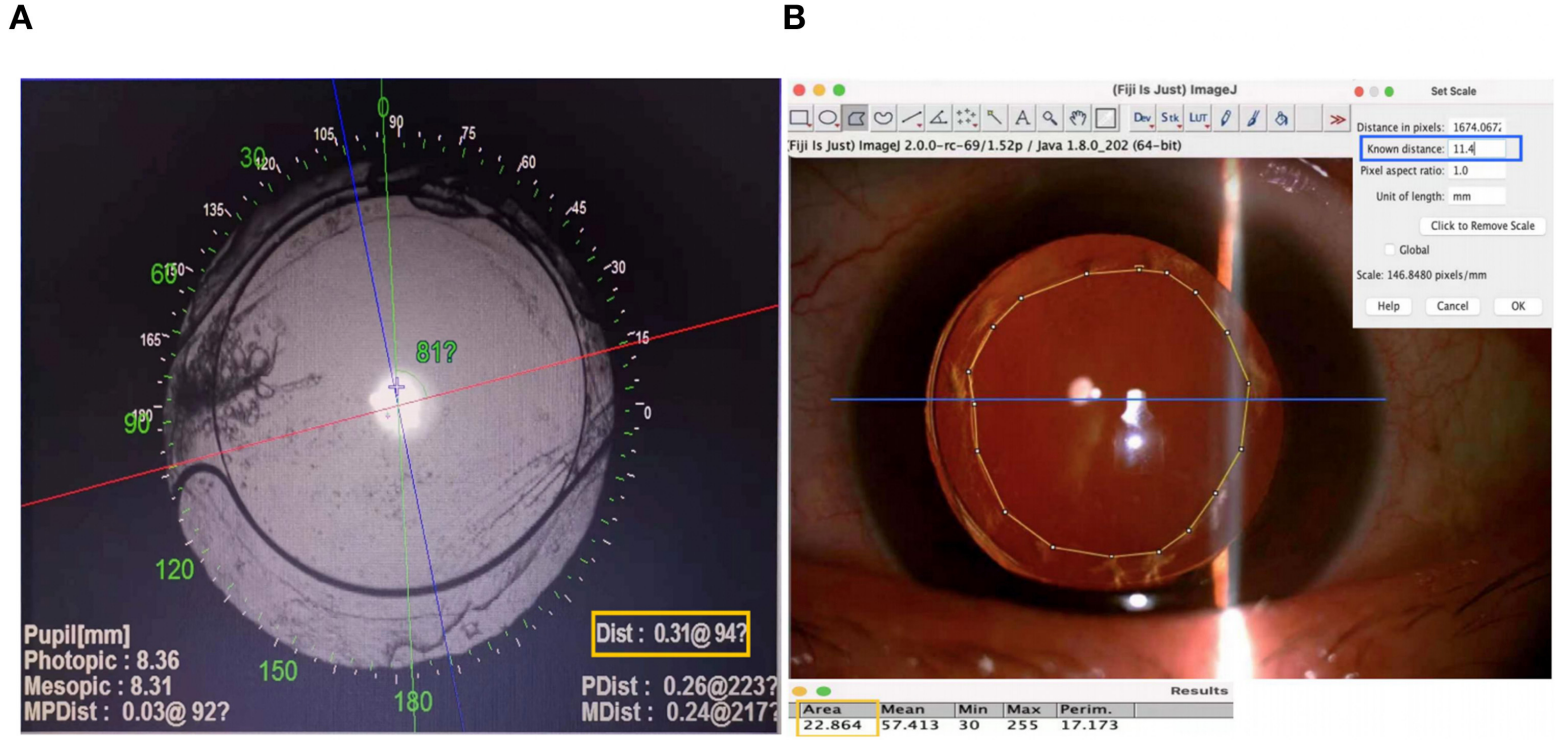
Methods for measuring intraocular lens (IOL) decentration and anterior capsular opening area. **(A)** The center of the visual axis refers to the point of intersection between the red and blue lines identified by the OPD Scan III aberrometer in the retroillumination analysis mode. The center of the IOL is determined according to the exposed edge of the optical region, which is indicated by the blue cross. The value and orientation of the overall decentration are shown in the yellow box. The horizontal and vertical decentrations are then figured out. **(B)** A scale is first set according to the corneal diameter of each patient. The blue line in the figure refers to the corneal diameter, and its known distance is entered into the corresponding input box, as shown in the blue box. Then, the anterior capsular opening (ACO) region is circled manually, and its area is figured out as results directly by Image J, as shown in the yellow box.

Intraocular lens decentration and tilt were obtained with the OPD-Scan III aberrometer (Nidek Co, Ltd., Gamagori, Japan) after the pupil was dilated until the edge of the IOL optics was visible. According to our previous study (10), the center of the visual axis was identified by the instrument in the retroillumination analysis mode, and the center of the IOL was determined based on the edge of its optical region. The overall decentration was defined as the distance from the center of the IOL to the center of the visual axis. The horizontal and vertical decentration were then determined using both the value and orientation of the overall decentration (Figure 1A). The tilt of the IOL was reported as an intraocular tilt datum. The OPD-Scan III aberrometer also provided intraocular high-order aberrations (HOAs) at 4 and 6 mm pupil diameters.

To measure the area of anterior capsular opening (ACO), images were taken using a Topcon slit lamp connected to a digital camera (Topcon, Tokyo, Japan). The ACO area was measured by Image J (National Institutes of Health, Bethesda, MD, USA). In brief, the ACO region was circled manually, and the value of the ACO area was figured out with a scale set according to each patient's corneal diameter, which was measured by Pentacam HR (Oculus Optikgeraete GmbH, Wetzlar, Germany) pre-operatively (Figure 1B).

### Statistical Analysis

All statistical analyses were performed using SPSS version 22 (IBM Corp., Chicago, IL, USA). Continuous data are presented as mean ± *SD*. The comparisons of continuous variables were assessed using Student's *t*-test between two groups. Categorical variables were compared using the χ^2^ test. Spearman's correlation analyses were used to analyze relationships between discontinuous variables. Backward stepwise multivariate linear regression analysis was performed to identify the factors that influenced overall and vertical decentration for Groups A and B, which included age, sex, eye laterality, AL, IOL weight, and ACO area as independent factors and IOL decentration as dependent factors, with adjustment of the interaction between AL and ACO or IOL weight. *P*-values <0.05 were considered statistically significant.

## Results

### Patient Characteristics

The characteristics of all included patients are shown in Table 1. No statistically significant differences were found in age, sex, operated eye, AL, IOL power, pre-operative UDVA, and CDVA between the two IOL groups (all *P* > 0.05). Significantly greater IOL weights were found in Group B compared to Group A (*P* < 0.001). Post-operative UDVA and CDVA did not show significant differences between the two groups at 3 years after surgery (both *P* > 0.05).

**Table 1:** Patient characteristics.

	**Group A (*N* = 86)**	**Group B (*N* = 119)**	***P*-value**
Age (years)	64.15 ± 8.52	63.92 ± 8.11	0.778
Sex (male/female)	35/51	52/67	0.668
Eye (right/left)	42/44	61/58	0.732
Axial length (mm)	29.44 ± 2.52	29.18 ± 2.18	0.450
IOL degree (D)	8.37 ± 6.12	8.73 ± 5.93	0.670
IOL weight (mg)	25.71 ± 4.62	28.31 ± 2.01	<0.001*
Pre-UDVA (logMAR)	1.10 ± 0.58	1.13 ± 0.52	0.448
Pre-CDVA (logMAR)	0.88 ± 0.53	0.94 ± 0.63	0.343
Post-UDVA (logMAR)	0.76 ± 0.42	0.69 ± 0.40	0.383
Post-CDVA (logMAR)	0.32 ± 0.44	0.30 ± 0.37	0.498

CDVA, Corrected distance visual acuity; D, diopter; IOL, Intraocular lens; logMAR, Logarithm of the minimal angle of resolution; N, Number of eyes; UDVA, Uncorrected distance visual acuity. Data are mean ± SD. *P <0.05.

### Post-Operative HOAs

**Figure 2:**
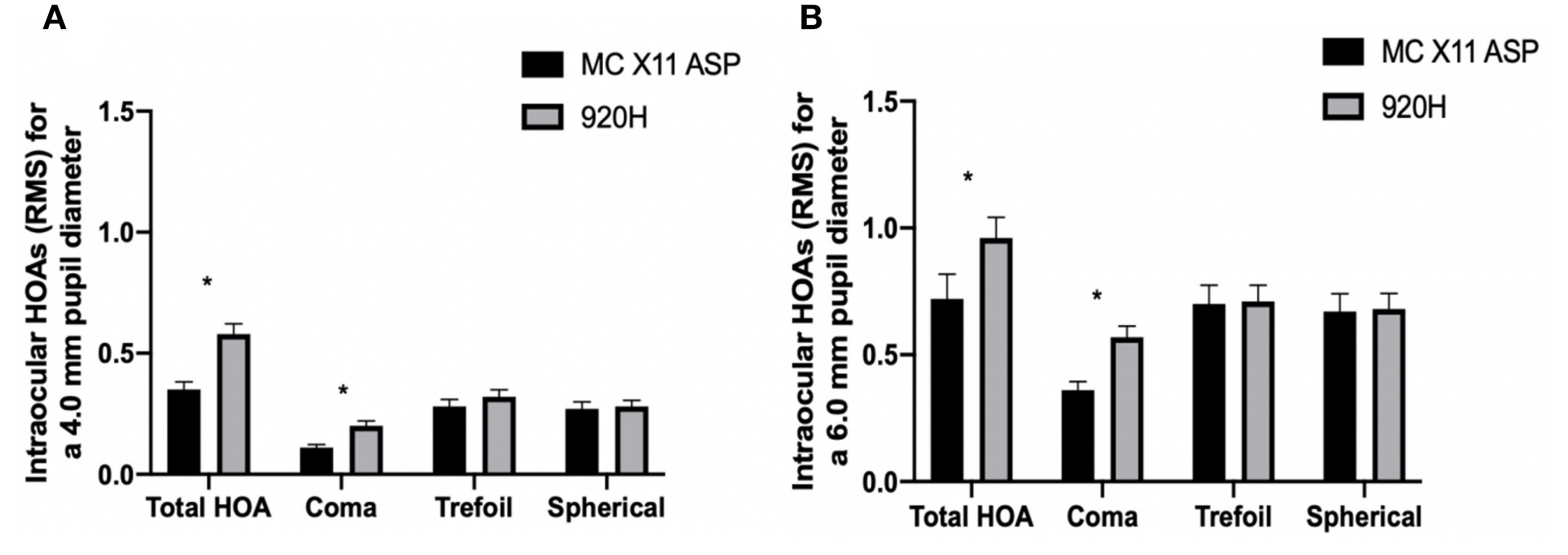
Intraocular high-order aberrations of Group A and Group B at 3 years after surgery. Between-group differences for intraocular aberrations at 4 mm **(A)** and 6 mm **(B)** pupil diameters. *P < 0.05. HOAs, higher-order aberrations; RMS, root mean square. Error bars represent the SEM.

In terms of intraocular HOAs, the total HOAs at both 4 and 6 mm pupil diameters were significantly greater in Group B compared to those in Group A (Group B vs. Group A: 4 mm pupil: 0.58 ± 0.46 μm vs. 0.35 ± 0.3 μm, *P* = 0.04; 6 mm pupil: 0.96 ± 0.89 μm vs. 0.72 ± 0.92 μm, *P* = 0.048; Figure 2). At both 4 and 6 mm pupil diameters, the coma aberrations were significantly greater in Group B compared to the Group A (Group B vs. Group A: 4 mm pupil: 0.2 ± 0.23 μm vs. 0.11 ± 0.12 μm, *P* = 0.047; 6 mm pupil: 0.57 ± 0.47 μm vs. 0.36 ± 0.32 μm, *P* = 0.044; Figure 2).

### Influence of IOL Weight on Long-Term IOL Decentration and Tilt

At 3 years after surgery, no significant differences were identified between the two groups for horizontal decentration and tilt (both *P* > 0.05). However, Group B presented significantly greater overall and vertical decentration than Group A (Group B vs. Group A: overall: 0.33 ± 0.21 mm vs. 0.24 ± 0.14 mm, respectively, *P* < 0.001; vertical: −0.19 ± 0.3 mm vs. −0.06 ± 0.18 mm, respectively, *P* < 0.001).

Table 2 shows the comparisons of long-term stability between the two IOL groups according to different ranges of IOL power. In the low and medium IOL power subgroups, Group B had significantly greater weight, and correspondingly greater overall and vertical decentration than Group A (all *P* < 0.05). While in the high IOL power subgroup, Group A showed slightly greater weight than Group B (*P* < 0.05), but no between-group difference was found for overall and vertical decentration (both *P* > 0.05). Moreover, no significant difference was found for horizontal decentration or tilt between the two IOL groups, regardless of the IOL power ranges (all *P* > 0.05).

**Table 2:** Comparisons of long-term intraocular lens (IOL) stability between Groups A and B with 3 different IOL power ranges.

**IOL power range**		**Group A**	**Group B**	**P value**
≥-5 to <5 D	*N* (%)	32 (46.4%)	37 (53.6%)	–
(low IOL power)	IOL weight (mg)	21.54 ± 1.81	26.16 ± 0.37	<0.001*
	Overall decentration (mm)	0.17 ± 0.14	0.31 ± 0.23	0.002*
	Vertical decentration (mm)	−0.00 ± 0.20	−0.14 ± 0.35	0.045*
	Horizontal decentration (mm)	−0.02 ± 0.24	−0.06 ± 0.28	0.589
	Intraocular tilt (μm)	0.77 ± 0.44	0.72 ± 0.52	0.579
≥5 to <14 D	*N* (%)	29 (33.3%)	58 (66.6%)	–
(medium IOL power)	IOL weight (mg)	24.96 ± 2.37	28.41 ± 1.11	<0.001*
	Overall decentration (mm)	0.24 ± 0.10	0.32 ± 0.20	0.013*
	Vertical decentration (mm)	−0.07 ± 0.16	−0.18 ± 0.28	0.018*
	Horizontal decentration (mm)	0.05 ± 0.24	−0.03 ± 0.38	0.630
	Intraocular tilt (μm)	0.81 ± 1.10	0.62 ± 0.68	0.186
14 D or greater	N (%)	25 (51.0%)	24 (49.0%)	–
(high IOL power)	IOL weight (mg)	31.90 ± 1.08	31.38 ± 0.49	0.035*
	Overall decentration (mm)	0.34 ± 0.14	0.38 ± 0.21	0.432
	Vertical decentration (mm)	−0.14 ± 0.16	−0.27 ± 0.28	0.051
	Horizontal decentration (mm)	−0.11 ± 0.17	−0.15 ± 0.34	0.652
	Intraocular tilt (μm)	0.57 ± 0.43	0.74 ± 1.12	0.175

D, diopter; IOL, Intraocular lens; N, Number of eyes. Data are mean ± SD. *P < 0.05.

**Figure 3:**
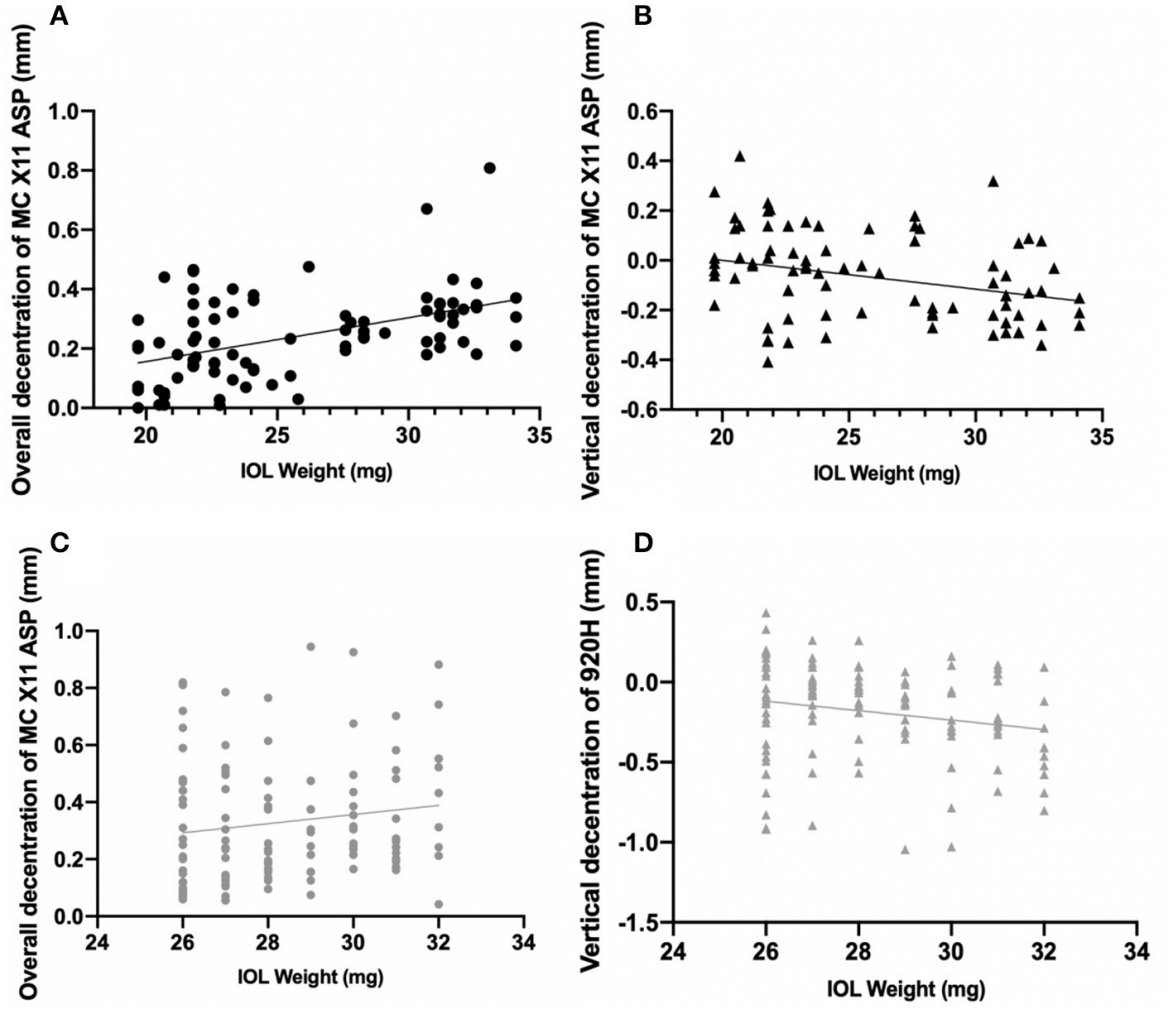
Correlations between decentration of intraocular lenses and IOL weight. **(A)** The black line indicates the overall decentration was positively correlated with IOL weight in Group A (*r* = 0.471, *P* < 0.001). **(B)** The black line indicates the vertical decentration was negatively correlated with IOL weight in Group A (*r* = 0.234, *P* = 0.01). **(C)** The gray line indicates the overall decentration was positively correlated with IOL weight in Group B (*r* = 0.234, *P* = 0.01). **(D)** The gray line indicates the vertical decentration was negatively correlated with IOL weight in Group B (*r* = −0.193, *P* = 0.036).

In either Group A or Group B, overall decentration was positively correlated with IOL weight (*r* = 0.471, *P* < 0.001, Figure 3A; *r* = 0.192, *P* = 0.037, Figure 3C, respectively), while vertical decentration was negatively correlated with IOL weight (*r* = −0.312, *P* = 0.003, Figure 3B; *r* = −0.2, *P* = 0.03, Figure 3D, respectively).

As to the ACO area, Group B presented a significantly larger ACO area than Group A (Group B vs. Group A: 20.33 ± 4.09 mm vs. 17.82 ± 6.69 mm, *P* < 0.001).

### Factors Influencing Long-Term IOL Decentration

In Group A, both greater overall and inferior decentration were significantly associated with greater IOL weight (β = 0.015, *P* < 0.001 and β = −0.012, *P* = 0.004, respectively). However, in Group B, greater overall decentration and inferior decentration was associated with greater IOL weight (β = 0.036, *P* = 0.001 and β = −0.054, *P* = 0.001, respectively) and longer AL (β = 0.041, *P* < 0.001 and β = −0.052, *P* < 0.001, respectively) after adjustment for the interactions between AL and ACO or IOL weight.

## Discussion

As the prevalence of high myopia rapidly increases, cataract surgery in highly myopic eyes has posed a great challenge for ophthalmologists worldwide (1), associated with more post-operative complications compared to emmetropic eyes (6). The IOL instability occurs more often in highly myopic eyes, which impairs the post-operative visual performance (10, 13, 14). Several IOL features associated with IOL stability have been reported, but the influence of IOL weight on post-operative long-term stability was not investigated. In this study, we compared the long-term IOL stability of MC X11 ASP and 920H IOLs in highly myopic eyes and found the heavier 920H IOL presented significantly greater overall and inferior decentration than the MC X11 ASP IOL, especially in low and medium IOL power subgroups. Meanwhile, the MC X11 ASP IOL performed better in terms of intraocular HOAs.

Based on this study, it could be inferred that greater IOL weight may lead to more severe long-term IOL decentration in highly myopic eyes. Studies have shown that the larger capsular bag in highly myopic eyes contributes to worse IOL stability (11, 12, 15). Nowadays, MC X11 ASP and 920H IOLs have been thought to be more suitable for highly myopic eyes (16–18). However, the IOL weight differs greatly between these two IOLs. Given that highly myopic eyes have weak zonular strength (9), we speculated that the long-term effect of the gravity of heavier IOLs may increase the burden of zonules, further resulting in reduced IOL stability. Yet, the influence of IOL weight on long-term IOL stability in highly myopic eyes was rarely studied. In the current study, the heavier 920H IOL presented greater long-term decentration in highly myopic eyes. For either the MC X11 ASP or 920H IOL, more severe long-term IOL decentration was associated with heavier IOL weight. Furthermore, the multivariate linear regression analysis with the adjustment for the interactions between AL and IOL weight, which was made separately in the two groups, also showed the association between IOL weight and long-term IOL decentration. These results demonstrated the influence of IOL weight on long-term IOL stability in highly myopic eyes shall not be ignored. Long-term post-operative follow-up is essential for highly myopic cataract patients.

The weight of IOL varies with power, resulting in different degrees of long-term IOL decentration after cataract surgery. In this study, we found that the heavier 920H IOL showed more severe long-term decentration than the MC X11 ASP IOL in highly myopic eyes, particularly when IOL power was <14 D. This is probably because IOLs with quite low power are always implanted in eyes with longer ALs and relatively weaker zonular strength. Therefore, for extremely myopic eyes, a lighter IOL may be more beneficial to the long-term IOL stability after surgery.

Another clinical significance of our study lies in providing an answer to the question of whether capsular tension rings (CTRs) are suitable to be implanted in highly myopic eyes. CTRs are commonly used to maintain post-operative integrity and stability of the capsular bag in order to stabilize IOL position (19, 20). However, in eyes with lower power of IOLs, that is, in extremely long eyes, the zonular strength is relatively weaker. The combined implantation of CTRs may aggravate the burden of gravity on the zonules and further impair the long-term IOL stability. Thus, CTRs should be used with caution, particularly for extremely myopic eyes.

Moreover, haptic design may partly affect long-term IOL stability. Our previous study showed capsular contraction syndrome occurred more frequently in the highly myopic population (6). The capsular bag starts to shrink 1 week after the operation and persists in contraction in the long run (21). The asymmetrical pressure from the contraction can affect the decentration and tilt of the IOL (22). In this study, the heavier 920H IOL showed greater inferior decentration, but its stronger AVH haptic design contributed to less anterior capsular contraction, thereby reducing the long-term IOL decentration caused by anterior capsular contraction. Conversely, the 920H IOL still showed greater decentration in the case of less anterior capsular contraction, which nevertheless highlights the influence of the IOL weight on the long-term IOL stability in highly myopic eyes.

Worse IOL stability can lead to higher intraocular HOAs, which consequently affects patients' visual quality (23). IOL decentration and tilt can lead to unexpected photopic symptoms including glare, halos, and mono diplopia (23, 24). A positive linear correlation was observed between IOL decentration and coma (24). Ocular coma-like aberrations caused by decentration and tilt of IOLs can result in myopic shift and oblique astigmatism, which are difficult to correct with regular glasses (25–27). In the current study, we observed that the 920H IOL presented greater decentration, and accordingly, eyes implanted with the 920H IOLs showed higher total HOA and coma at both 4.0 and 6.0 mm pupil diameters than those with MC X11 ASP IOLs.

Notably, this study proposed an interesting topic, which is to explore the potential relationship between IOL weight and IOL long-term decentration in highly myopic eyes. Since this article is a retrospective study, prospective, randomized, and multicentered trials with controlled confounding factors would be needed to better investigate this topic. In terms of the long-term clinical performance of two different IOLs, the significant difference lies in HOAs rather than post-operative visual acuity. A follow-up study about subjective perceptions could be an excellent idea for further research.

In conclusion, heavier IOLs may lead to greater long-term decentration as well as higher HOAs post-operatively in highly myopic eyes. Our results may provide helpful advice for cataract surgeons on IOL selection among highly myopic patients, especially among extremely myopic patients, and thus improve their long-term visual satisfaction.

## Data Availability Statement

The raw data supporting the conclusions of this article will be made available by the authors, without undue reservation.

## Ethics Statement

This retrospective study was conducted at the Eye and Ear, Nose, and Throat Hospital, Fudan University, Shanghai, in accordance with the tenets of the Declaration of Helsinki. All procedures were approved by the Institutional Review Board of the Eye and Ear, Nose, and Throat Hospital. Signed informed consents were obtained from all participants before cataract surgery for the use of their clinical data. The study was affiliated with the Shanghai High Myopia Study (registered at www.clinicaltrials.gov, accession number: NCT03062085). The patients/participants provided their written informed consent to participate in this study.

## Author Contributions

YC was responsible for conception and design, analysis and interpretation of data, and writing the manuscript. JM was responsible for conception and design, analysis and interpretation of data, critical revision of the manuscript, and supervision. QL and KC were responsible for data collection. LW was responsible for the critical revision of the manuscript and supervision. YL and XZ were responsible for conception and design, technical support, critical revision of the manuscript, and obtaining funding. All authors contributed to the article and approved the submitted version.

## Funding

This article was supported by research grants from the National Natural Science Foundation of China (Nos. 82122017, 81870642, 81970780, and 81670835), Science and Technology Innovation Action Plan of Shanghai Science and Technology Commission (No. 19441900700, and No. 21S31904900), Clinical Research Plan of Shanghai Shenkang Hospital Development Center (No. SHDC2020CR4078, and No. SHDC12019X08), Double-E Plan of Eye & Ear, Nose, and Throat Hospital (SYA202006), Shanghai Municipal Key Clinical Specialty Program (shslczdzk01901), and the Fudan University Outstanding 2025 Program.

## Conflict of Interest

The authors declare that the research was conducted in the absence of any commercial or financial relationships that could be construed as a potential conflict of interest.

## Publisher's Note

All claims expressed in this article are solely those of the authors and do not necessarily represent those of their affiliated organizations, or those of the publisher, the editors and the reviewers. Any product that may be evaluated in this article, or claim that may be made by its manufacturer, is not guaranteed or endorsed by the publisher.

## Supplementary Material

Supplementary Table 1 The baseline data of Group A and Group B. Between the two groups, no statistically significant differences were found in age, sex, operated eye, AL, IOL power, pre-operative UDVA and CDVA (all *P* > 0.05). Significantly greater IOL weights were found in Group B compared to Group A (*P* < 0.001). Post-operative UDVA and CDVA did not show significant differences between the two groups at 3 years after surgery (both *P* > 0.05).
