# Supplementary material for: Influence of IOL Weight on Long-Term IOL Stability in Highly Myopic Eyes
Source: Front Med (Lausanne). 2022 Apr 11;9:835475. doi: 10.3389/fmed.2022.835475 (PMC9035698; doi:10.3389/fmed.2022.835475)
Supplement: Supplementary Table 1 — The baseline data of Group A and Group B. Between the two groups, no statistically significant differences were found in age, sex, operated eye, AL, IOL power, pre-operative UDVA and CDVA (all P > 0.05). Significantly greater IOL weights were found in Group B compared to Group A (P < 0.001). Post-operative UDVA and CDVA did not show significant differences between the two groups at 3 years after surgery (both P > 0.05). [file Table_1.pdf]

MC X11 ASP (HumanOptics AG)		920H (Rayner)	
Power (D)	Weight (mg)	Power (D)	Weight (mg)
-5.0	27.8	-5.0	26.0
-4.0	26.8	-4.0	26.0
-3.0	25.8	-3.0	26.0
-2.0	24.8	-2.0	26.0
-1.0	23.8	-1.0	26.0
0.0	22.8	0.0	26.0
1.0	21.8	1.0	26.0
2.0	20.7	2.0	26.0
3.0	19.7	3.0	26.0
4.0	20.5	4.0	27.0
5.0	21.2	5.0	27.0
6.0	21.9	6.0	27.0
7.0	22.6	7.0	27.0
8.0	23.3	8.0	27.0
9.0	24.1	9.0	28.0
10.0	24.8	10.0	28.0
10.5	25.5	10.5	28.0
11.0	26.2	11.0	29.0
11.5	26.9	11.5	29.0
12.0	27.6	12.0	29.0
12.5	28.3	12.5	29.0
13.0	29.1	13.0	30.0
13.5	29.7	13.5	30.0
14.0	30.2	14.0	30.0
14.5	30.7	14.5	30.0
15.0	31.2	15.0	31.0
15.5	31.7	15.5	31.0
16.0	32.1	16.0	31.0
16.5	32.6	16.5	31.0
17.0	33.1	17.0	32.0
17.5	33.6	17.5	32.0
18.0	34.1	18.0	32.0
18.5	27.2	18.5	32.0
19.0	27.6	19.0	33.0
19.5	28.0	19.5	33.0
20.0	28.4	20.0	33.0
20.5	28.8	20.5	33.0
21.0	29.2	21.0	34.0
21.5	29.6	21.5	34.0

22.0	30.0	22.0	34.0
22.5	30.3		
